# Investigating Microenvironmental Regulation of Human Chordoma Cell Behaviour

**DOI:** 10.1371/journal.pone.0115909

**Published:** 2014-12-26

**Authors:** Priya Patel, Courtney Brooks, Ayesh Seneviratne, David A. Hess, Cheryle A. Séguin

**Affiliations:** 1 Department of Anatomy and Cell Biology, Schulich School of Medicine & Dentistry, The University of Western Ontario, London, Ontario, Canada; 2 Department of Physiology and Pharmacology, Schulich School of Medicine & Dentistry, The University of Western Ontario, London, Ontario, Canada; 3 Robarts Research Institute, The University of Western Ontario, London, Ontario, Canada; University of Pécs Medical School, Hungary

## Abstract

The tumour microenvironment is complex and composed of many different constituents, including matricellular proteins such as connective tissue growth factor (CCN2), and is characterized by gradients in oxygen levels. In various cancers, hypoxia and CCN2 promote stem and progenitor cell properties, and regulate the proliferation, migration and phenotype of cancer cells. Our study was aimed at investigating the effects of hypoxia and CCN2 on chordoma cells, using the human U-CH1 cell line. We demonstrate that under basal conditions, U-CH1 cells express multiple CCN family members including *CCN1, CCN2, CCN3* and *CCN5*. Culture of U-CH1 cells in either hypoxia or in the presence of recombinant CCN2 peptide promoted progenitor cell-like characteristics specific to the notochordal tissue of origin. Specifically, hypoxia induced the most robust increase in progenitor-like characteristics in U-CH1 cells, including increased expression of the notochord-associated markers *T, CD24, FOXA1, ACAN* and *CA12*, increased cell growth and tumour-sphere formation, and a decrease in the percentage of vacuolated cells present in the heterogeneous population. Interestingly, the effects of recombinant CCN2 peptide on U-CH1 cells were more pronounced under normoxia than hypoxia, promoting increased expression of *CCN1*, *CCN2*, *CCN3* and *CCN5*, the notochord-associated markers *SOX5*, *SOX6*, *T*, *CD24*, and *FOXA1* as well as increased tumour-sphere formation. Overall, this study highlights the importance of multiple factors within the tumour microenvironment and how hypoxia and CCN2 may regulate human chordoma cell behaviour.

## Introduction

Chordomas are rare, malignant and locally invasive tumours that originate in bones of the skull and spine, and are thought to arise from cellular remnants of the embryonic notochord. These tumours occur most commonly at the base of the skull (32%) and sacrococcygeal region (29%), and less frequently in cervical, thoracic and lumbar vertebrae [1], [2]. The cancer typically affects one in one million people each year in the United States, with the median age of diagnosis being 49 years for skull-based chordomas and 69 years for sacral-based chordomas [2].

During embryonic development, notochord cells act as tissue-specific progenitor cells that give rise to the nucleus pulposus of the intervertebral disc [3], [4]; however, during spine formation and notochord segmentation some of these notochord cells get trapped within the vertebral bone and are referred to as benign notochord remnants. Since these benign notochord remnants give rise to chordomas, it has been suggested that factors associated with the regulation of embryonic notochord development may likewise be associated with malignant transformation and the development of chordomas [5]. For example, studies have demonstrated that brachyury (T), a transcription factor necessary for the formation and maintenance of the notochord [6], is amplified in sporadic chordomas and duplicated in familial chordomas [7], [8], [9]. In addition to T, other transcription factors have been implicated in notochord development such as the SOX (SRY-type high mobility group box) family members SOX5, SOX6 and SOX9 [10], [11] and the forkhead box proteins A1 and A2 (FOXA1 and FOXA2) [12].

There are a limited number of studies that have examined the effects of the tumour microenvironment on human chordoma cell biology. Two important components of the tumour microenvironment are the oxygen concentration and matricellular proteins, including CCN proteins. Hypoxic conditions (usually between 1–3% O_2_ but vary depending on the type of tumour [13]) often result from inadequate oxygen supply to the tumour, which can be caused by low oxygen tension in arterial blood, limited ability for blood to carry oxygen, reduced tissue perfusion or inconsistencies in blood flow diffusion [14]. Normally, these conditions are detrimental to cells, but cancer cells adapt to the hypoxic environment. For example, under hypoxia prostate cancer cells show increased cell proliferation [15], and prostate [15], breast [16] and colon [17] cancer cells display increased migration compared to cells cultured under normoxia. In addition, studies have shown that hypoxia can promote stem and progenitor cell properties in various cancers including glioma, glioblastoma and ovarian cancer [18], [19].

Connective tissue growth factor (CCN2; formerly known as CTGF) is part of the CCN family of matricellular proteins. CCN2 is expressed in many tissues including the notochord [20] and nucleus pulposus [21] and is an important regulator of notochord development [22]. CCN2 also has a role in cancer cell biology and has been shown to promote cell proliferation, colony formation, migration and angiogenesis in a cell type-specific manner [23]. CCN2 has also been shown to modulate stem and progenitor cell properties; mesenchymal stem cells treated with recombinant CCN2 (rCCN2) demonstrated reduced differentiation, whereas the addition of rCCN2 to hepatic progenitor cells promoted hepatocytic differentiation [24], [25].

The specific effects of hypoxia and CCN2 on chordoma cells are largely unknown. Studies have demonstrated that a large volume of chordoma tumours are hypoxic [26] and that CCN2 is a direct downstream target of T in chordoma [27]. In this study, we sought to better understand the role of the tumour microenvironment by specifically investigating the effects of hypoxia and CCN2 on the regulation of chordoma cells using the human U-CH1 cell line [28]. Through characterization of gene expression and functional analyses, we demonstrate that exposure of U-CH1 cells to hypoxic conditions or recombinant CCN2 peptide promoted progenitor-like characteristics specific to the notochordal tissue of origin. Interestingly, exposure of chordoma cells to hypoxia induced more pronounced changes in *in vitro* cell behaviour than did exposure of cells to CCN2. Furthermore, we found an additive effect on the expression of a subset of notochord progenitor markers by U-CH1 cells when CCN2 and hypoxia were combined in the cell microenvironment.

## Materials and Methods

### Cell culture

All experiments were conducted using the human U-CH1 chordoma cell line [28]. Cells were maintained using previously established protocols in IMDM/RPMI (4∶1) (Invitrogen, Life Technologies) supplemented with 10% fetal bovine serum (Invitrogen, Life Technologies) (chordoma media) on 0.1% gelatin coated cell culture plates. U-CH1 cells were maintained in monolayer cultures and passaged by enzymatic dissociation at a ratio of 1∶3–1∶6 for cell expansion. Recombinant human CCN2 (PHG0286, Life Technologies) was resuspended in 0.1% bovine serum albumin in PBS. This 11.2 kDa peptide fragment corresponds to amino acids 253–349 of the full length human CCN2 and contains the cysteine knot domain. U-CH1 cells were treated with 50, 100 or 200 ng/mL rCCN2 in IMDM/RPMI (4∶1) with 5% FBS. All cell culture was carried out at 37°C in a humidified atmosphere of 5% CO_2_ in either normoxic (20% O_2_) or hypoxic (2% O_2_) conditions. Cells were maintained for at least 1–2 passages in either normoxia or hypoxia before experiments were performed.

### Immunocytochemistry

Confluent monolayer cultures of U-CH1 cells cultured on glass-bottom culture dishes (MatTek Corporation) were fixed in 4% paraformaldehyde (37°C) for 10 min. Cells were washed three times in 0.1% Triton X-100 in PBS (PBST) and then blocked using 5% species-specific serum in PBST for 1 h at room temperature. Cells were then incubated overnight at 4°C in 5% species-specific serum in PBST containing primary antibodies against human CCN1 (1∶200; sc13100, Santa Cruz Biotechnology), CCN2 (1∶200; sc14939, Santa Cruz Biotechnology), brachyury (1∶400; sc17743, Santa Cruz Biotechnology) or HIF-1α (1∶100; 07–628, Millipore). Cells were washed and incubated with fluorescence-conjugated secondary antibodies (donkey anti-goat IgG (A11055), goat anti-rabbit IgG (A11008) or donkey anti-mouse IgG (A11001), Life Technologies). For nuclear staining, 1 ug/mL Hoechst 33258 (Sigma-Aldrich) was added in PBST for 10 min. IgG and secondary only controls were run in parallel with experimental samples to detect non-specific binding. Images were acquired using a Leica Microsystems DM16000B fluorescent microscope and DFC360FX camera.

### Real-time PCR

Cells were harvested directly in TRIzol Reagent (Life Technologies). Total RNA was extracted according to the manufacturer's protocol and quantified using a NanoDrop 2000 spectrophotometer (Thermo Scientific). For each sample, 0.5 µg RNA was used for cDNA synthesis (iScript, Bio-Rad Laboratories). Real-time PCR was performed using the Bio-Rad CFX384 system. PCR reactions were run in triplicate using 27 ng of cDNA per reaction and 310 nM forward and reverse primers (sequences provided in Table 1) with 2X SsoFast EvaGreen Supermix (Bio-Rad Laboratories). The PCR program was an initial 2 min at 95°C followed by 40 cycles of 10 sec at 95°C, and 20 sec annealing/elongation at 60°C. Gene expression was normalized relative to a standard curve (1/5 serial dilution with initial input of 0.1 µg/µL) made from a mixture of cDNA generated from U-CH1 cells, human foreskin fibroblast cells and human embryonic stem cells in a 2∶2∶1 ratio.

**Table 1:** Primer sequences used for real-time PCR gene expression analysis.

Gene Name	Primer Sequence (5′ to 3′)
*CCN1*	Fwd- ACCGCTCTGAAGGGGATCT Rev- ACTGATGTTTACAGTTGGGCTG
*CCN2*	Fwd- TCCCAAAATCTCCAAGCCTA Rev- GTAATGGCAGGCACAGGTCT
*CCN3*	Fwd- AGTGATGGTCATTGGGACCTG Rev- CCTCTGGTAGTCTTCAGCTCC
*CCN5*	Fwd- GCGACCAACTCCACGTCTG Rev- TCCCCTTCCCGATACAGGC
Brachyury *(T)*	Fwd- TGAGACCCAGTTCATAGCGG Rev- TGCTGGTTCCAGGAAGAAGC
*CD24*	Fwd- CTCCTACCCACGCAGATTTATTC Rev- AGAGTGAGACCACGAAGAGAC
*ACAN*	Fwd- TGAGGAGGGCTGGAACAAGTACC Rev- GGAGGTGCTAATTGCAGGGAACA
*FOXA1*	Fwd- GCAATACTCGCCTTACGGCT Rev- TACACACCTTGGTAGTACGCC
*CA12*	Fwd- TGGCATTCTTGGCATCTGTA Rev- TTGGTGGCTGGCTTGTAAAT
*SOX5*	Fwd- CAGCCAGAGTTAGCACAATAGG Rev- CTGTTGTTCCCGTCGGAGTT
*SOX6*	Fwd- TACCTCTACCTCACCACATAAGC Rev- ACATCGGCAAGACTCCCTTTG
*SOX9*	Fwd- AGCGAACGCACATCAAGAC Rev- CTGTAGGCGATCTGTTGGGG
*HIF1A*	Fwd- ATCCATGTGACCATGAGGAAATG Rev- TCGGCTAGTTAGGGTACACTTC
*VEGFA*	Fwd- AGGGCAGAATCATCACGAAGT Rev- AGGGTCTCGATTGGATGGCA
*GLUT1*	Fwd- GGCCAAGAGTGTGCTAAAGAA Rev- ACAGCGTTGATGCCAGACAG

### Cell growth assay

U-CH1 cells were plated at a density of 3,430 cells/cm^2^ in chordoma media or rCCN2 media and maintained under either normoxic or hypoxic conditions. Every 24 h over 8 days, cells were trypsinized and counted in triplicate with a hemocytometer on a phase contrast light microscope using Trypan Blue (Gibco) to exclude non-viable cells.

### Cell migration assay

U-CH1 cells were plated in chordoma media at 25,714 cells/cm^2^ and maintained under either normoxic or hypoxic conditions. After 24 h, media was changed to IMDM/RPMI (4∶1) with 0.2% FBS to permit cell survival but minimize cell proliferation. After 24 h, an artificial “scratch wound” was created using a pipette tip. Cells were imaged at 0, 12, 24, 36 and 48 h at the location of the scratch. Images were exported to ImageJ software and the polygon selection tool was used to calculate the area of new cell migration at each time point. The percentage of wound closure was calculated using the formula [(Area_t-0h_ – Area_t-Δh_)/Area_t-0h_] ×100%, as previously reported [29].

### Sphere formation assay

U-CH1 cells were plated using standard protocols for tumour-sphere formation, at 1,500 cells/well in 24-well non-adherent plates (Nunc, Thermo Scientific) and grown for 10 days in serum-free DMEM/F12 (Life Technologies) supplemented with B27 (1∶50 dilution), bFGF (20 ng/mL), EGF (20 ng/mL) and 1% methylcellulose (R&D Systems). For rCCN2 experiments, the above media was supplemented with 100 ng/mL rCCN2. Cells were fed every other day and spheres (defined as ≥50 µm) were counted after 10 days. The efficiency of sphere formation (number of spheres per 1000 cells) was calculated, as previously described [30].

### Flow cytometry

U-CH1 cells were harvested from monolayer by enzymatic dissociation in 0.25% Trypsin and resuspended in 10% FBS in PBS. Cells were resuspended at 1×10^6^ cells/mL in Aldefluor buffer (Stemcell Technologies). 2 µL/mL Aldefluor reagent (Stemcell Technologies) was added to determine aldehyde dehydrogenase (ALDH) activity, with an ALDH inhibitor (diethylaminobenzaldehyde (DEAB)) as a negative control (Stemcell Technologies). Samples were incubated for 30 min, pelleted by centrifugation (470 *g*, 7 min) and resuspended in Aldefluor buffer (1×10^6^ cells in 200 µL) with fluorochrome-conjugated antibodies specific to human CD90 (555596, BD Biosciences), CD105 (562380, BD Biosciences), CD133 (130090826, Miltenyi Biotec) or IgG controls for 30 min. After incubation, ALDH and cell surface expression was assessed in a minimum of 100,000 cells/treatment group, using a LSR II flow cytometer (BD Biosciences) at the London Regional Flow Cytometry Facility, and analyzed on FlowJo software program. U-CH1 cells were characterized based on side scatter using cluster gating, as previously described [31].

### Statistical analysis

All data was collected and statistical analysis was performed using GraphPad Prism 6.0 Software. Data was analyzed using either unpaired Student's t test (between 2 groups), 1-way ANOVA (between ≥3 groups; comparing one condition) or 2-way ANOVA (between ≥3 groups; comparing two conditions) with either a Tukey's multiple comparisons test or Dunnett's test to examine differences compared to a control group.

## Results

### Expression of T and CCN proteins in U-CH1 cells

**Figure 1:**
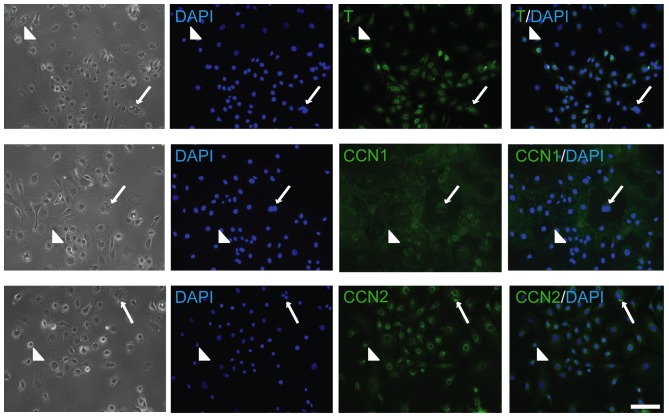
Localization of T, CCN1 and CCN2 in U-CH1 cells. Representative images demonstrating the localization of T, CCN1 and CCN2 in U-CH1 cells. For each, protein expression was detected in both vacuolated (arrow) and non-vacuolated (arrowhead) cells in the heterogenous cell population. (n = 3; N = 3; scale bar = 100 µm).

The expression of the known chordoma marker brachyury (T) and the matricellular proteins CCN1 and CCN2 were first assessed in U-CH1 cells. In keeping with previous reports [32], immunolocalization demonstrated expression and nuclear localization of T in the majority of U-CH1 cells (both vacuolated and non-vacuolated cell morphologies) (Fig. 1). We also detected expression of CCN1 and CCN2 in both vacuolated and non-vacuolated U-CH1 cells, localized to the perinuclear and cytoplasmic regions of the cells (Fig. 1).

### Effects of hypoxia on U-CH1 gene expression

**Figure 2:**
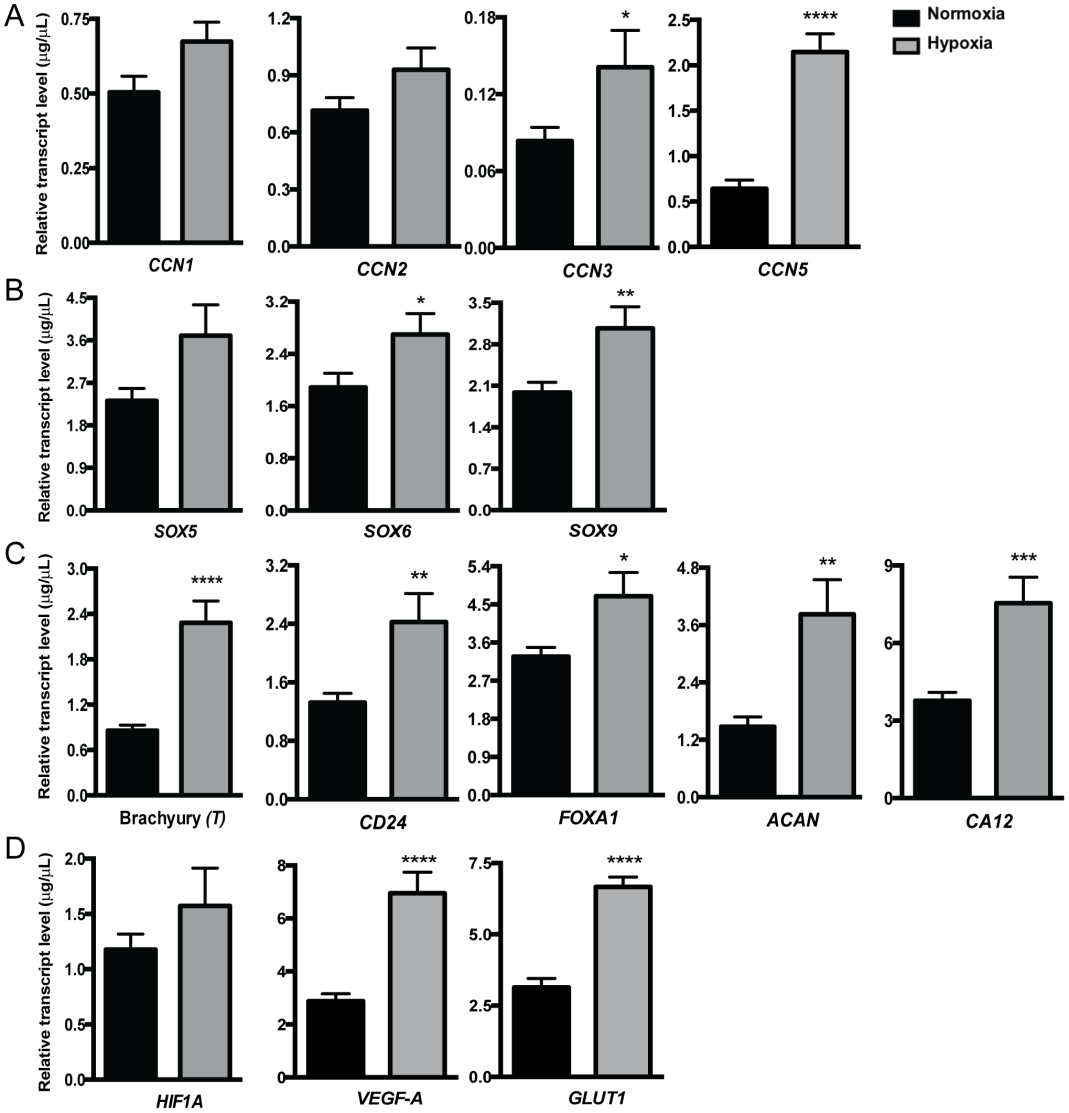
Effect of hypoxia on gene expression in U-CH1 cells. Gene expression was assessed in U-CH1 cells maintained for at least 2 passages in either normoxic (20% O_2_) or hypoxic (2% O_2_) conditions by real-time PCR. Expression of (**A**) members of the CCN family (*CCN1, CCN2, CCN3, CCN5*), (**B**) members of the SOX transcription factor family (*SOX5, SOX6, SOX9*), (**C**) notochord markers (*CD24, T, AGG, FOXA1, CA12*) and (**D**) hypoxia inducible factor (*HIF1A*) and its downstream targets (*VEGF-A, GLUT1*) were investigated. Data is presented as the mean ±SEM, changes assessed using unpaired Student's t test; * = P≤0.05, ** = P≤0.01, *** = P≤0.001, **** = P≤0.0001 (n = 3; N = 3–4).

To determine the effects of changes in oxygen levels in the cell microenvironment on the chordoma cell gene expression profile, U-CH1 cells were grown in either normoxic (20% O_2_) or hypoxic (2% O_2_) conditions and a panel of genes were investigated. We first assessed expression of CCN family members, *CCN1* [33], *CCN2* [34], [35], *CCN3* [36] and *CCN5* [37], [38] which have been implicated in modulating cell proliferation, cell adhesion, angiogenesis, cell migration, metastasis and epithelial-mesenchymal transition in a variety of cancers. While no change in *CCN1* or *CCN2* expression was detected, culture of U-CH1 cells in hypoxia promoted a significant increase in the expression of *CCN3* and *CCN5* compared to normoxia (Fig. 2A). We next investigated the expression of SOX family members *SOX5, SOX6* and *SOX9*. Culture of cells in hypoxia induced a significant increase in *SOX6* and *SOX9* expression in U-CH1 cells compared to cells maintained in normoxia (Fig. 2B). To determine if the expression of early notochord progenitor markers was altered in U-CH1 cells under hypoxic conditions, we examined the expression of *T, CD24, FOXA1, ACAN* and *CA12* (Fig. 2C). Culture of U-CH1 cells in hypoxia induced a significant up-regulation in the expression of all these markers, with *T* and *CA12* having the most significant increase (P<0.001) followed by *CD24* and *ACAN* (P<0.01) and then *FOXA1* (P<0.05). Lastly, to determine if HIF-1α was activated in U-CH1 cells under hypoxic conditions, we interrogated the expression of *HIF1A* and its downstream targets *VEGF-A* and *GLUT1* (Fig. 2D). Although no change was detected in the expression of *HIF1A*, chordoma cells demonstrated a significant increase in the expression of both *VEGF-A* and *GLUT1* in hypoxia compared to cells maintained in normoxia.

### Changes in CCN1 localization in hypoxia

**Figure 3:**
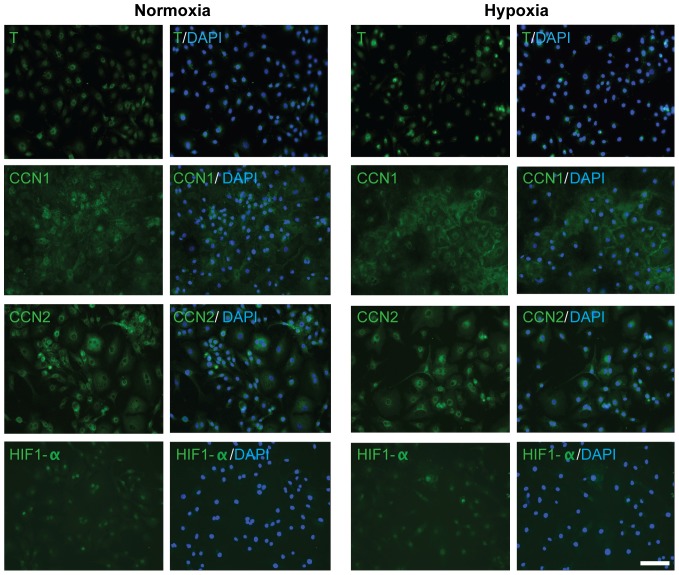
Expression and localization of T, CCN1, CCN2 and HIF-1α in U-CH1 cells maintained under normoxic (20% O_2_) or hypoxic (2% O_2_) conditions. Representative images demonstrating changes in the localization of target proteins in U-CH1 cells cultured in hypoxia. CCN1 demonstrates diffuse nuclear and cytoplasmic localization under normoxia but is concentrated to the cytoplasmic and cell periphery in cells under hypoxia. HIF-1α demonstrates primarily nuclear localization. CCN2 is localized to the nuclear and cytoplasmic region, and T is localized to the nuclear region under both normoxia and hypoxia. (n = 3; N = 3; scale bar  = 100 µm).

Protein localization in U-CH1 cells maintained in either hypoxic or normoxic conditions was directly compared (Fig. 3). In normoxia, perinuclear and diffuse cytoplasmic CCN1 staining was detected in U-CH1 cells. In contrast, CCN1 appeared to be concentrated at the cell periphery in U-CH1 cells maintained in hypoxia. HIF-1α was detected primarily in the nucleus of U-CH1 cells under both normoxia and hypoxia. No changes were detected in the localization of either CCN2 or T in U-CH1 cells maintained in hypoxia compared to cells maintained in normoxia.

### Hypoxia promotes U-CH1 cell growth and sphere formation but reduces cell migration

**Figure 4:**
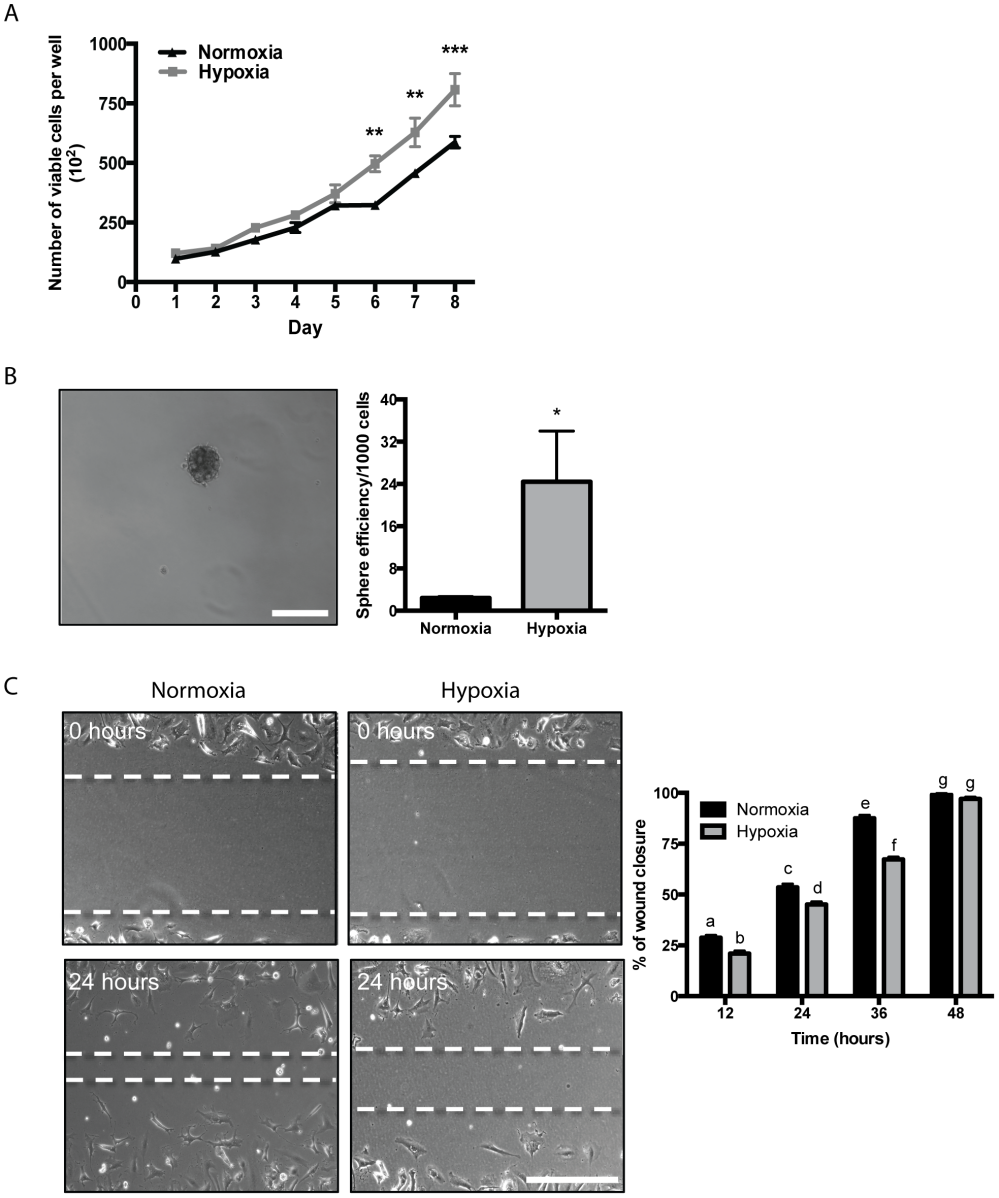
Cell growth, migration and tumour-sphere formation in U-CH1 cells in normoxic (20% O_2_) or hypoxic (2% O_2_) conditions. (**A**) U-CH1 cells were counted every 24 h over 8 days and demonstrate increased growth in hypoxia *versus* normoxia at days 6–8 (n = 3; N = 3). (**B**) U-CH1 cells were grown in suspension and spheres were counted after 10 days. Cells maintained under hypoxic conditions demonstrate a significant increase in sphere formation compared to cells maintained in normoxic conditions (N = 3; scale bar = 250 µm). (**C**) U-CH1 cells were plated and an artificial “scratch wound” was created. Cells were imaged at 12, 24, 36 and 48 h and the area of new cell migration borders was determined. Maintenance of cells in hypoxia significantly decreased cell migration at 12, 24 and 36 h compared to cells in normoxia at the same time-point (n = 4; N = 4). (2-way ANOVA with Tukey's test (A and C); Unpaired Student's t test (B); * = P≤0.05; ** = P≤0.001; *** = P≤0.0001; scale bar = 250 µm).

To investigate cell growth, U-CH1 cells were counted every 24 h over 8 days in either normoxic or hypoxic conditions (Fig. 4A). U-CH1 cells demonstrated increased growth in hypoxia compared to cells maintained in normoxia at days 6, 7 and 8. In addition to changes at specific time points, there was a significant increase in the rate of cell growth under hypoxia compared to normoxia, as determined by linear regression analysis. To investigate stem-like properties, tumour-sphere formation assays were conducted with U-CH1 cells under normoxic or hypoxic conditions (Fig. 4B). Quantification of the efficiency of sphere formation following 10 days of culture demonstrated a significant 10-fold increase in sphere formation in U-CH1 cells maintained in hypoxia compared to cells maintained in normoxia. To evaluate changes in U-CH1 cell migration in hypoxia compared to normoxia, a “scratch wound” assay was conducted and cell migration was assessed over 48 h (Fig. 4C). U-CH1 cells maintained in hypoxia demonstrated a significant decrease in cell migration at 12, 24 and 36 h compared to cells maintained in normoxia. This difference was no longer apparent at 48 h, as the majority of the wound appeared to be closed under both conditions. Taken together, these findings suggest that hypoxia could be promoting progenitor cell-like properties in U-CH1 cells.

### rCCN2 peptide promotes *ACAN* and *COL2A1* gene expression in U-CH1 cells

Previous studies demonstrated the up-regulation of *ACAN* and *COL2A1* gene expression in nucleus pulposus cells following stimulation with rCCN2 [39], [40]. To ensure bioactivity of the 11.2 kDa rCCN2 peptide in UCH-1 cells, we quantified expression of *ACAN* and *COL2A1* in UCH-1 cells following 24 h treatment with 50, 100 or 200 ng/mL rCCN2. Treatment of cells with 100 ng/mL rCCN2 resulted in a significant increase in *ACAN* and *COL2A1* expression under both normoxia and hypoxia. Treatment of cells with 200 ng/mL rCCN2 increased the expression of *COL2A1*, however this effect was only significant under normoxia (S1 Fig.). A concentration of 100 ng/mL rCCN2 was therefore used in all subsequent experiments.

### Effects of rCCN2 peptide on U-CH1 gene expression under normoxia and hypoxia

**Figure 5:**
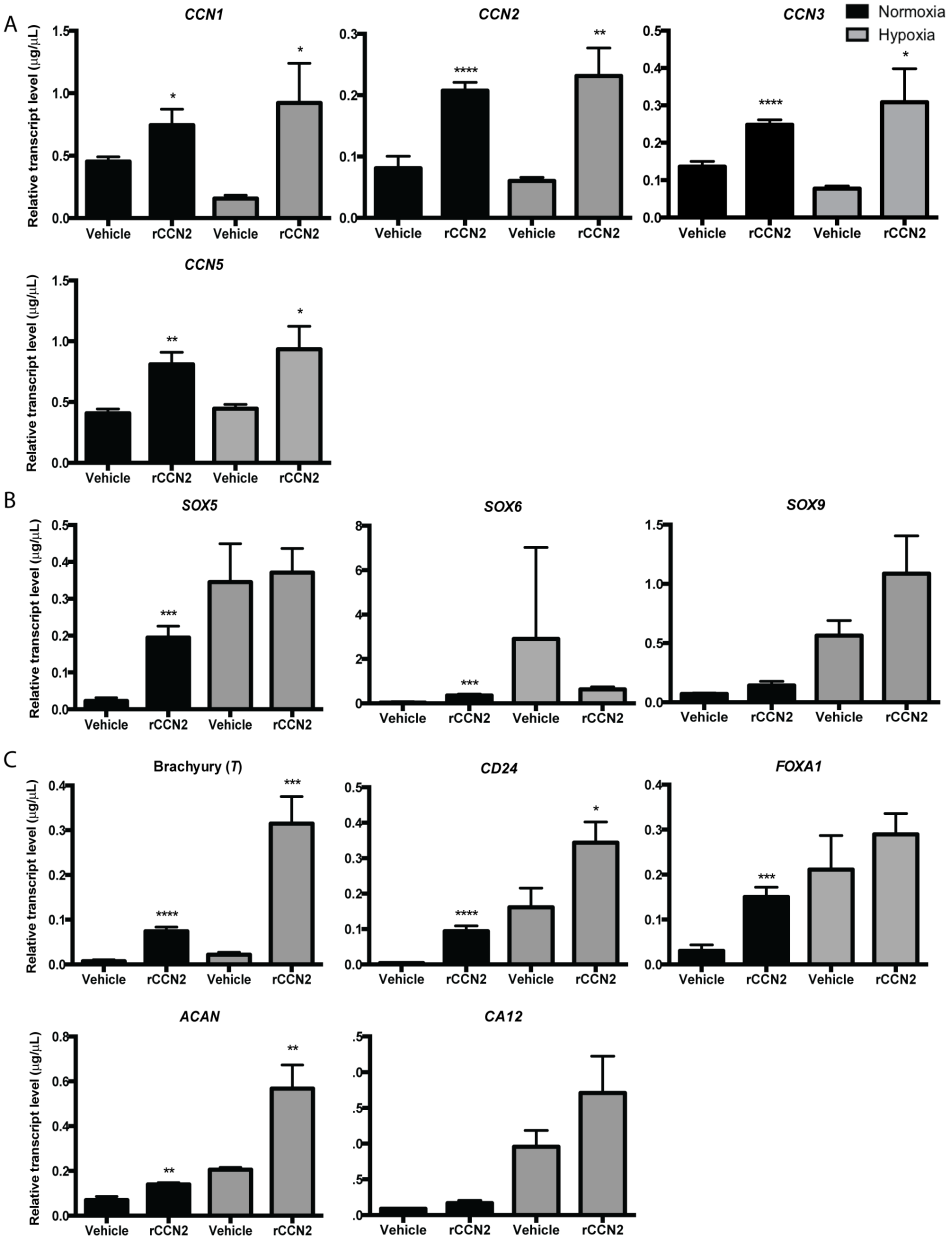
Effect of rCCN2 peptide on U-CH1 gene expression in normoxic (20% O_2_) or hypoxic (2% O_2_) conditions. Expression of (**A**) members of the CCN family (*CCN1, CCN2, CCN3, CCN5*), (**B**) members of the SOX transcription factor family (*SOX5, SOX6, SOX9*) and (**C**) notochord markers *(T, CD24, FOXA1, ACAN, CA12)* were investigated in U-CH1 cells treated with 100 ng/mL of rCCN2 for 24 h in either normoxic or hypoxic conditions. Data is presented as the mean ±SEM, assessed using an Unpaired Student's t test; n = 3, N = 3; * = P≤0.05, ** = P≤0.01, *** = P≤0.001, **** = P≤0.0001.

To determine the effects of CCN2 on chordoma cells, U-CH1 cells were treated with rCCN2 peptide under either normoxic or hypoxic conditions for 24 h, and a panel of genes was investigated. We first assessed the expression of *CCN1, CCN2, CCN3* and *CCN5* and found a significant increase in all of these genes with the addition of rCCN2 under either normoxia or hypoxia (Fig. 5A). We also assessed the expression of SOX family members and found a significant increase in *SOX5* and *SOX6* gene expression with the addition of rCCN2 under normoxia, but no change was detected when rCCN2 was added to cells in hypoxia (Fig. 5B). Lastly, we investigated the expression of early notochord markers (Fig. 5C). U-CH1 cells treated with rCCN2 in normoxia demonstrated a significant increase in the expression of *T, CD24, FOXA1* and *ACAN* compared to untreated controls. In hypoxia, treatment of U-CH1 cells with rCCN2 induced a significant increase in the expression of *T, CD24* and *ACAN* compared to untreated controls. Interestingly, rCCN2 and hypoxia induced an additive response in the expression of a subset of genes in U-CH1 cells, specifically *T, CD24* and *ACAN*. Together, these data suggest that rCCN2 peptide may be promoting a progenitor cell-like phenotype in U-CH1 cells, with the effects being more robust under normoxia than hypoxia.

### rCCN2 peptide increases U-CH1 tumour-sphere formation but does not increase cell growth

**Figure 6:**
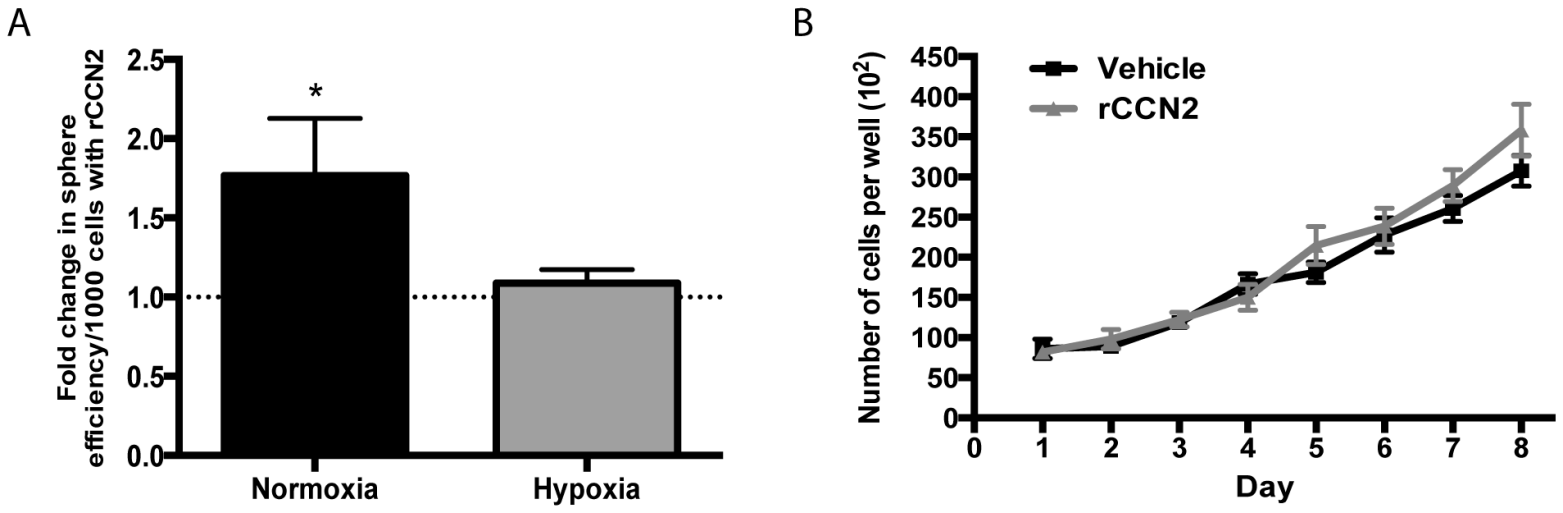
Sphere formation and cell growth in U-CH1 cells treated with rCCN2. (**A**) Sphere formation was assessed in U-CH1 cells treated with rCCN2 (100 ng/mL) in either normoxic or hypoxic conditions. The efficiency of sphere formation was assessed at day 10 as the number of spheres formed per 1000 cells. Data is presented as fold change from vehicle control (set to 1; indicated by dashed line) for cells maintained in either normoxic or hypoxic culture conditions. Treatment of cells with rCCN2 induced a significant increase in sphere formation compared to vehicle control when cells were maintained in normoxia (N = 3–4). (**B**) U-CH1 cells were maintained in normoxic conditions and treated with 100 ng/mL of rCCN2 or vehicle control and cell number was quantified every 24 h over 8 days. No change in cell growth was detected following treatment with rCCN2 compared to vehicle control (n = 3, N = 3). Unpaired Student's t test (A); 2-way ANOVA with Tukey's test (B); * = P≤0.05; scale bar = 250 µm.

Sphere formation was assessed for U-CH1 cells grown in either normoxia or hypoxia with the addition of rCCN2 for 10 days. U-CH1 cells demonstrated a significant 1.7-fold increase in tumour-sphere formation with the addition of rCCN2 in normoxia compared to vehicle control. In contrast, treatment of U-CH1 cells with rCCN2 in hypoxia did not alter sphere formation compared to vehicle control (Fig. 6A). We then assessed U-CH1 cell growth in the presence of rCCN2 in normoxia, as these culture conditions more significantly induced changes in U-CH1 cell properties. Over 8 days, treatment of U-CH1 cells with rCCN2 did not alter cell growth (Fig. 6B). We also performed a linear regression analysis and found no changes in the rate of cell growth between U-CH1 cells treated with rCCN2 and vehicle control.

### Maintenance of U-CH1 cells in hypoxia leads to a decrease in the number of vacuolated cells

**Figure 7:**
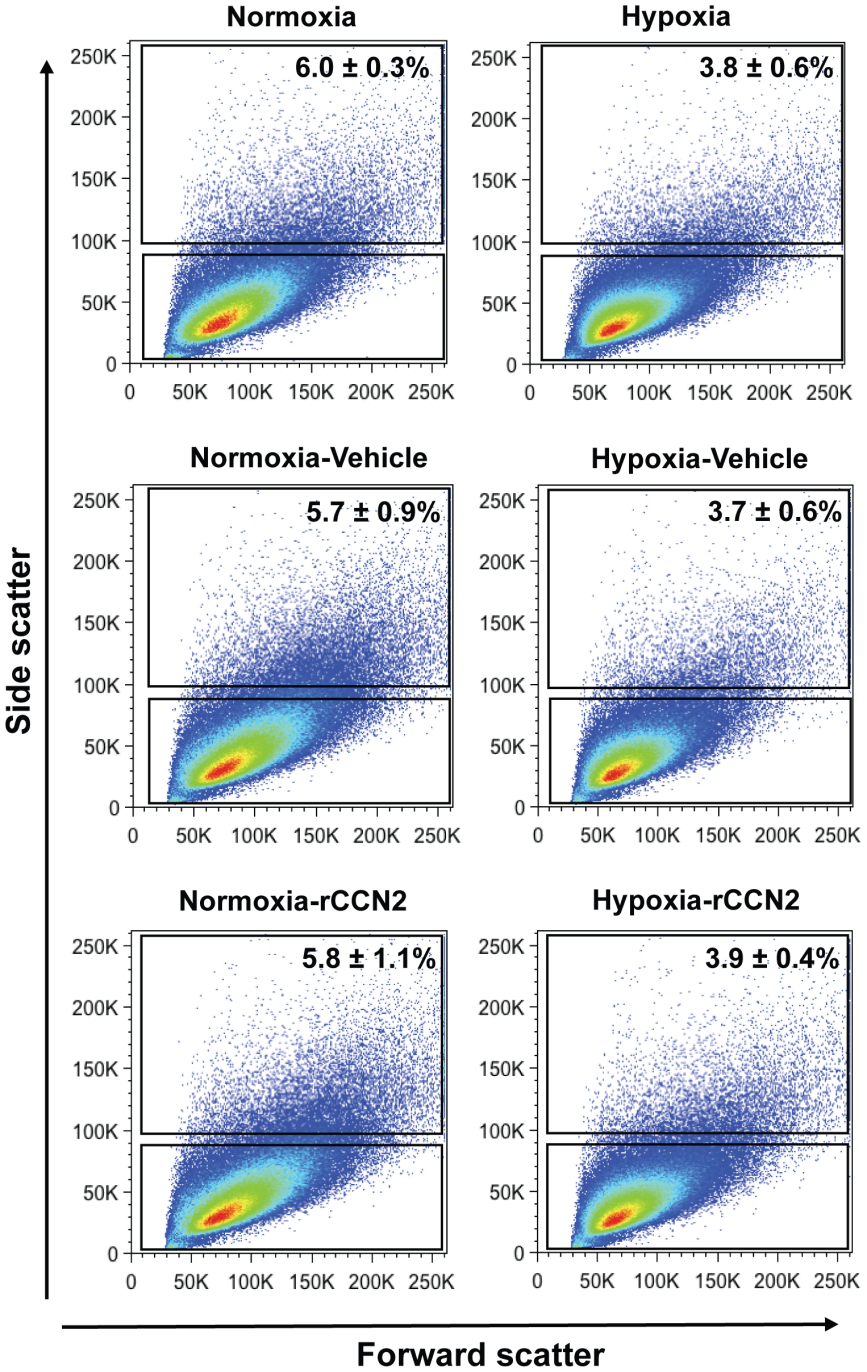
Side scatter distribution of U-CH1 cells maintained in normoxic (20% O_2_) or hypoxic (2% O_2_) conditions, with or without rCCN2. Cluster gating was used to determine the intracellular complexity of UCH-1 cells. Cells demonstrated a significant decrease in the number of vacuolated cells (side scatter high) under hypoxia compared to normoxia with minimal changes detected following culture of cells for 6 days in the presence of 100 ng/mL rCCN2 (N = 3).

To determine if culture of U-CH1 cells in hypoxia or the treatment of cells with rCCN2 peptide affected the morphological characteristics of the heterogeneous chordoma cell population, we assessed intracellular complexity of cells by flow cytometry. Recent studies have suggested that non-vacuolated chordoma cells represent the progenitor cells within the heterogeneous population, giving rise to the characteristic larger vacuolated chordoma cells [41]. We first assessed U-CH1 cells maintained in either normoxia or hypoxia and found a significant decrease in the percentage of vacuolated U-CH1 cells (high side scatter) in hypoxia compared to cells maintained in normoxia (3.8±0.6% *vs*. 6.0±0.3%, respectively). Interestingly, treatment of cells with rCCN2 over 6 days did not alter the percentage of vacuolated U-CH1 cells in either normoxia (5.7±0.9% vehicle *vs*. 5.8±1.1% rCCN2) or hypoxia (3.7±0.6% vehicle *vs*. 3.9±0.4% rCCN2) (Fig. 7).

To investigate if a cancer stem cell population could be detected in U-CH1 cells, the expression of CD133 and the activity of aldehyde dehydrogenase (ALDH), common markers of cancer stem cells [42], [43], [44], [45], [46], were examined in cells maintained in either normoxia or hypoxia, with our without the addition of rCCN2. We detected minimal expression of CD133 (>0.01% positive cells) and minimal ALDH activity (>0.02% ALDH^hi^ cells) in U-CH1 cells under all culture conditions examined. We further investigated expression of the cell surface markers CD90 and CD105 which were previously detected in U-CH1 cells [47] and act as differentiation markers in mesenchymal stromal cells [48]. No significant change in the percentage of CD90^+^ or CD105^+^ cells was detected when U-CH1 cells were maintainted in normoxic or hypoxic conditions, with or without the addition of rCCN2 (S2 Fig.).

## Discussion

The tumour microenvironment is composed of a variety of different cell types such as cancer cells, stromal fibroblasts, endothelial cells, whose function is modulated by oxygen levels, the extracellular matrix and secreted proteins, including CCN proteins [49]. The current study investigated the effects of hypoxia and the matricellular protein CCN2 on U-CH1 cells, as there is strong indication that these factors could be involved in the regulation of chordoma cell biology [26], [27]. In this study, we demonstrate that U-CH1 cells express multiple CCN proteins and show that their expression is regulated by the chordoma microenvironment. Using a combination of gene expression analysis and *in vitro* functional assays, we found that hypoxia had the greatest ability to promote progenitor-like properties in U-CH1 cells compared to treatment of cells with rCCN2 peptide. In addition, we demonstrated that the effects of rCCN2 and hypoxia were additive for the expression of a specific subset of notochord progenitor markers.

In this study, we demonstrate the expression of CCN matricellular proteins in U-CH1 cells through immunolocalization and gene expression analysis. To our knowledge, this is the first investigation of CCN protein expression in chordoma cells and suggests they could have a potential role as regulators of chordoma cell biology. Through gene expression analysis, we detected expression of *CCN1*, *CCN2*, *CCN3* and *CCN5* in U-CH1 cells under basal conditions, and demonstrate increased expression of *CCN3* and *CCN5* by chordoma cells in hypoxia. These findings are in keeping with previous studies that reported increased expression of CCN1 and CCN3 in human choriocarcinoma cells [50], increased expression of CCN1 in retinal vascular endothelial cells [51], and increased expression of CCN2 in primary tubular epithelial cells [52]cultured in hypoxia compared to cells cultured in normoxia. Interestingly, we also detected changes in the localization of CCN proteins in U-CH1 cells depending on the oxygen levels in their culture environment, including nuclear localization of both CCN1 and CCN2. Previous studies have reported similar nuclear localization of CCN proteins in cancer cells, including nuclear localization of CCN1 in prostate carcinoma [53] and breast cancer cells [54] and the nuclear localization of CCN2 in melanoma cells [55]. While the cellular function of CCN proteins within the nucleus remains unknown, the report of their nuclear localization in multiple studies suggests they may perform additional cellular functions. For example, a truncated form of CCN3 lacking the secretory signal peptide is directed to the nucleus and has been shown to exhibit negative transcriptional activity [56]. Similarly, after binding at the cell surface, CCN2 can be transported via endocytosis to the nucleus, although its function there remains unknown [57].

When U-CH1 cells were maintained in hypoxia, we found an increase in the expression of notochord markers (*T, CD24, FOXA1, ACAN* and *CA12*), tumour-sphere formation and cell growth, and a decrease in cell migration. Our findings are consistent with recent molecular profiling of chordoma cells that identified CD24 in a list of potential candidates for chordoma tumorigenesis [9]. There are currently opposing reports as to the effects of hypoxia on human chordoma cell growth [58], [59]. Our findings are consistent with those of Ostroumov *et al*., which reported an increase in cell growth in a primary human chordoma cells in hypoxia compared to normoxia. The decrease in cell migration detected in U-CH1 cells in hypoxia contrasts reports showing that hypoxia increases the migratory potential of various cancer cell types, including glioma [60], epithelial ovarian cancer [61], breast cancer [62] and gastric cancer cells [63]. It is tempting to speculate that this response may be related to properties of the notochordal cell type from which chordomas are derived, which adapt to survive within the hypoxic environment of the intervertebral disc during normal development [64]. Further validating a change in U-CH1 cell phenotype in hypoxia, we demonstrated a decrease in the number of vacuolated U-CH1 cells in hypoxia compared to normoxia. These findings are in keeping with a recent study suggesting that vacuolated cells represent a more differentiated cell type within the heterogeneous U-CH1 cell population [41]. Our findings are also in line with studies in other cancers which reported the promotion of stem and progenitor cell properties following culture of cells in hypoxia [19], [65].

In hypoxia, cells typically demonstrate increased activity of HIF-1α, a transcription factor that binds to hypoxia-response elements to activate the expression of genes that regulate proliferation, angiogenesis, metabolism, apoptosis and the maintenance of an undifferentiated state in cancer cells [66], [67], [68]. For example, vascular endothelial growth factor (VEGF) and glucose transporter 1 (GLUT1) are direct downstream targets of HIF-1α involved in angiogenesis and glycolysis, respectively [69]. Under normoxic conditions, the α subunit of the HIF-1α protein is rapidly degraded through hydroxylation of two key proline residues in the oxygen-dependent degradation domain [70]. However, under hypoxic conditions, HIF-1α becomes stabilized, translocates to the nucleus, and dimerizes with the aryl hydrocarbon receptor nuclear translocator (ARNT) binding to the HIF-1 consensus sequence to activate hypoxia-inducible genes. Recent studies have reported HIF-1α expression in the majority of chordoma samples examined, correlated with VEGF expression and tumour microvessel density [18], [71]. While we did not detect the induction of *HIF1A* gene expression in U-CH1 cells maintained in hypoxia, we detected a significant up-regulation of *VEGF-A* and *GLUT1* gene expression, suggesting increased HIF-1α activity in hypoxia.

Compared to the effects of hypoxia on U-CH1 cells, we found that treatment of cells with exogenous CCN2 induced the expression of fewer notochord markers (*T, CD24, FOXA1 and ACAN*) and SOX family members (*SOX5, SOX6*). Along with changes in gene expression, we found an increase in sphere formation but no changes in cell growth. Interestingly, we found that addition of rCCN2 peptide to cells grown in hypoxia induced even fewer changes in the expression of notochord progenitor markers, no change in the expression of SOX family members, and did not promote sphere formation. These differences in the effect of exogenous CCN2 treatment in normoxic or hypoxic conditions may be related to the cell-type specific effects of CCN proteins. We demonstrated that U-CH1 cells are more progenitor-cell like under hypoxia than in normoxia. In these conditions, cells may express a different profile of integrins, cell surface receptors or CCN binding proteins that mediate CCN2-dependent signalling. Furthermore, recent studies have established an intriguing direct feedback between HIF-1α and CCN2 in related cell types. In chondrocytes, HIF-1α directly interacts with the *Ccn2* promoter and the addition of rCCN2 increases HIF-1α mRNA and protein levels [72]. In nucleus pulposus cells, the loss of HIF-1α was reported to increase *CCN2* expression [73], [74], [75]. Based on these findings, we speculate that in addition to independent CCN2 and HIF-1α induced pathways, there could be interaction between HIF-1α and CCN2 in chordoma cells, such that rCCN2 is decreasing HIF-1α activity under hypoxia but promoting HIF-1α activity under normoxia.

Taken together, findings from this study highlight the importance of the tumour microenvironment in the regulation of human chordoma cell phenotype. We demonstrate that components of the microenvironment influence the chordoma cell phenotype and that cells respond to hypoxia and exogenous CCN2 by up-regulating progenitor cell-like properties. Although further studies are required to validate these findings in other chordoma cell lines or primary tumour cells, our findings suggest an intriguing commonality between the pathways associated with notochord development and those associated with tumour pathology. As has recently been suggested for other cancer types [76], morphogens and extracellular signals that regulate embryonic notochord development may also play key roles in establishing a microenvironment that promotes chordoma pathogenesis.

## Supporting Information

S1 Fig **Effect of rCCN2 on** ***ACAN*** **and** ***COL2A1*** **gene expression in U-CH1 cells maintained in normoxic (20% O_2_) or hypoxic (2% O_2_) conditions.** U-CH1 cells were treated with 50, 100 or 200 ng/mL of rCCN2 for 24 h and harvested for gene expression analysis. Treatment of cells with 100 ng/mL of rCCN2 promoted a significant increase in *ACAN* and *COL2A1* gene expression in cells under both normoxia and hypoxia. Data is presented as the mean ±SEM assessed using 1-way ANOVA with Dunnet's test; n = 3; N = 3; * = P≤0.05; ** = P≤0.001.
(TIF)

S2 Fig **Expression of cell surface markers CD90 and CD105 in U-CH1 cells with or without rCCN2 in normoxic (20% O_2_) or hypoxic (2% O_2_) conditions.** Percentage of CD90^+^ and CD105^+^ cells U-CH1 cells as detected by flow cytometry. Expression of CD90 and CD105 cells was detected in U-CH1 cells maintained under normoxia and hypoxia, with no significant change in the expression of these markers induced by either oxygen conditions or treatment with rCCN2 peptide. Data is presented as the mean ±SEM; N = 3.
(TIFF)
