# Supplementary figures and images for: Investigating Microenvironmental Regulation of Human Chordoma Cell Behaviour
Source: PLoS One. 2014 Dec 26;9(12):e115909. doi: 10.1371/journal.pone.0115909 (PMC4277432; doi:10.1371/journal.pone.0115909)

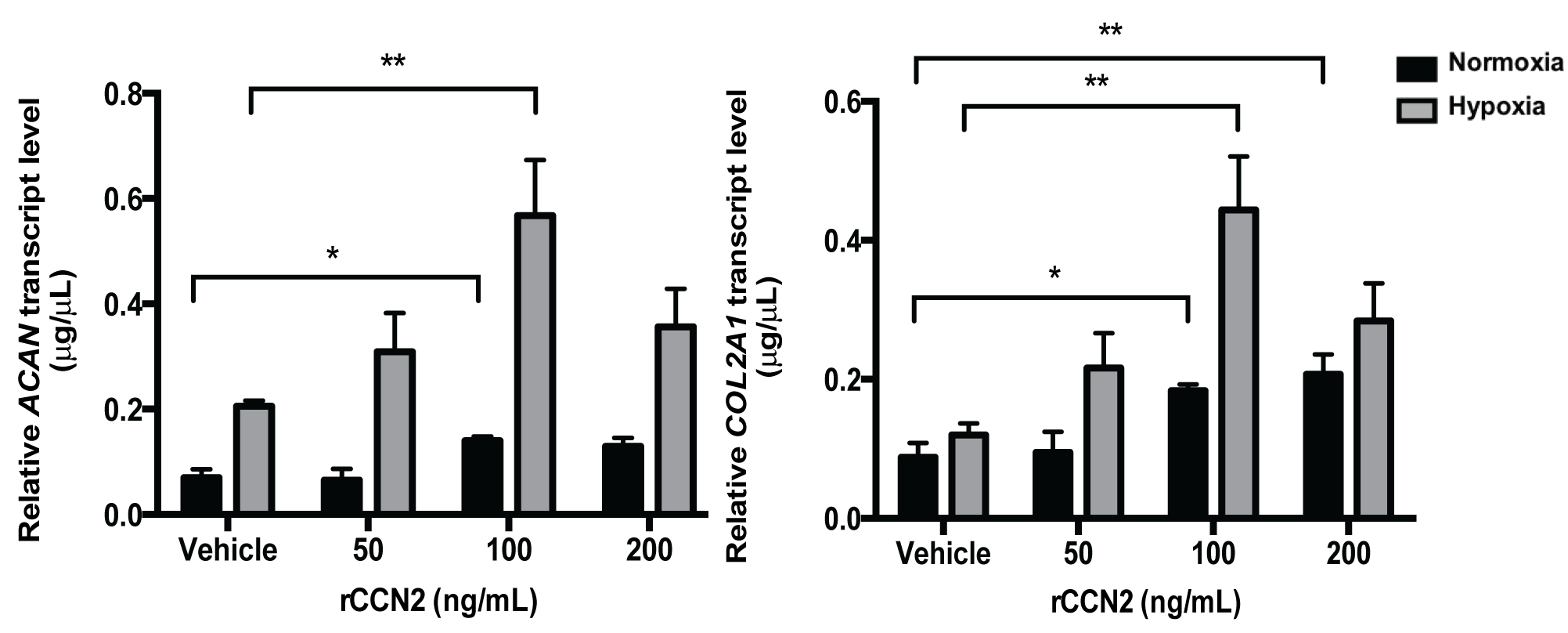

Supplement: S1 Fig — Effect of rCCN2 on ACAN and COL2A1 gene expression in U-CH1 cells maintained in normoxic (20% O2) or hypoxic (2% O2) conditions. U-CH1 cells were treated with 50, 100 or 200 ng/mL of rCCN2 for 24 h and harvested for gene expression analysis. Treatment of cells with 100 ng/mL of rCCN2 promoted a significant increase in ACAN and COL2A1 gene expression in cells under both normoxia and hypoxia. Data is presented as the mean ±SEM assessed using 1-way ANOVA with Dunnet's test; n = 3; N = 3; * = P≤0.05; ** = P≤0.001. (TIF) [file pone.0115909.s001.tif]

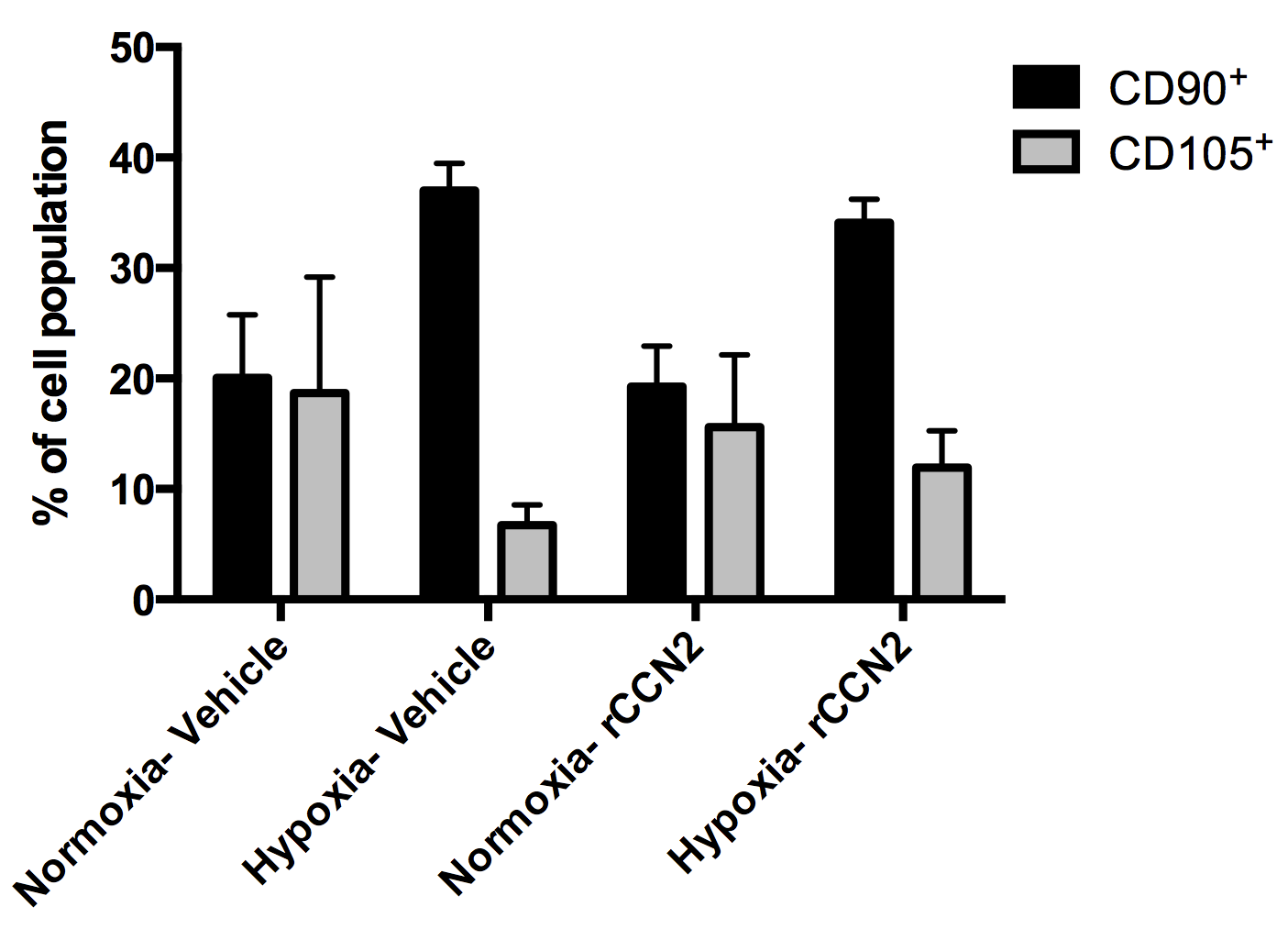

Supplement: S2 Fig — Expression of cell surface markers CD90 and CD105 in U-CH1 cells with or without rCCN2 in normoxic (20% O2) or hypoxic (2% O2) conditions. Percentage of CD90+ and CD105+ cells U-CH1 cells as detected by flow cytometry. Expression of CD90 and CD105 cells was detected in U-CH1 cells maintained under normoxia and hypoxia, with no significant change in the expression of these markers induced by either oxygen conditions or treatment with rCCN2 peptide. Data is presented as the mean ±SEM; N = 3. (TIFF) [file pone.0115909.s002.tiff]
